# A Biologically Inspired Approach to Frequency Domain Feature Extraction for EEG Classification

**DOI:** 10.1155/2018/9890132

**Published:** 2018-01-23

**Authors:** Nurhan Gursel Ozmen, Levent Gumusel, Yuan Yang

**Affiliations:** ^1^Department of Mechanical Engineering, Karadeniz Technical University, 61080 Trabzon, Turkey; ^2^Department of Physical Therapy and Human Movement Sciences, Feinberg School of Medicine, Northwestern University, Chicago, IL, USA

## Abstract

Classification of electroencephalogram (EEG) signal is important in mental decoding for brain-computer interfaces (BCI). We introduced a feature extraction approach based on frequency domain analysis to improve the classification performance on different mental tasks using single-channel EEG. This biologically inspired method extracts the most discriminative spectral features from power spectral densities (PSDs) of the EEG signals. We applied our method on a dataset of six subjects who performed five different imagination tasks: (i) resting state, (ii) mental arithmetic, (iii) imagination of left hand movement, (iv) imagination of right hand movement, and (v) imagination of letter “A.” Pairwise and multiclass classifications were performed in single EEG channel using Linear Discriminant Analysis and Support Vector Machines. Our method produced results (mean classification accuracy of 83.06% for binary classification and 91.85% for multiclassification) that are on par with the state-of-the-art methods, using single-channel EEG with low computational cost. Among all task pairs, mental arithmetic versus letter imagination yielded the best result (mean classification accuracy of 90.29%), indicating that this task pair could be the most suitable pair for a binary class BCI. This study contributes to the development of single-channel BCI, as well as finding the best task pair for user defined applications.

## 1. Introduction

The idea of people being able to control their brain rhythm by performing specific mental tasks constitutes the main research focus on electroencephalogram (EEG) based mental control tasks, which gave birth to the brain-computer interface (BCI) [1]. BCI provides the user, the communication, and control possibility that is independent of peripheral nerves and muscles [2]. A typical BCI system consists of four stages: signal acquisition, feature extraction, classification, and transformation to an output device [3]. To build a well-performing BCI, feature extraction is an important aspect. Several studies suggested that the use of an efficient feature extraction method may improve the final performances more than using an efficient classifier [4].

Despite the large numbers of feature extraction methods that have been developed for BCIs [5–14], the performances of current BCIs are still not satisfactory. Thus, the selection of efficient features is still a key challenge to be addressed [15, 16]. Due to the volume conduction effect and artefacts, EEG signal has a poor signal-to-noise ratio [17]. Commonly used feature extraction methods for BCIs, such as common spatial pattern (CSP) filter [18] and independent component analysis (ICA) [13], usually require multiple EEG channels for gathering enough information for precise decoding. Multichannel EEG recording reduces the portability of daily use BCI and therefore constitutes the main drawback for end users [19]. To address these problems, many methods have been proposed in the literature including electrode reduction algorithms and feature extraction methods based on a few electrodes [16, 19–24]. However, most of them either have high computational complexity [20–22] or are only suitable for specific motor imagery tasks [16, 19, 23, 24]. For online BCI applications, a quick response time is a key issue and thus the efficient feature extraction methods with low computational complexity and minimum number of channels are highly desirable [16, 24]. Moreover, many studies tested feature extraction methods and classification algorithms only on BCI competition datasets [16, 19, 21–24]. Although BCI competitions provide a useful platform for testing and comparing different algorithms, subject specific property of BCI may prevent transplantation of algorithm performance from one dataset to another [19]. Therefore, for a real BCI application, it is advantageous to conduct BCI studies including data recording, instead of only using BCI competition datasets [25, 26].

In this paper, a novel* biologically inspired* approach using single EEG channel is proposed to extract frequency domain features. Based on the new approach, we aim to improve the classification performance and reduce the number of electrodes required in EEG classification. Frequency band features are a golden standard for EEG classification since they represent the rhythmic neural activity within the different frequency bands [4, 8, 9]. Changes in these rhythms due to movement or imagination of various tasks provide useful features for binary or multiclass classification [4]. Thus, our method considers the biological information in the EEG signal, which is different from the existing purely data-driven approaches in BCIs. Moreover, using small number of channels for EEG classification is advantageous since it takes less preparation time and is highly preferred by the end users [27]. That is important for daily use BCIs [23]. To the best of our knowledge, there are only few single-channel EEG studies in the literature and all of them are limited to a specific mental task (e.g., an imagination of foot movement or a visual task) [28–31].

Different from previous studies, our method greatly reduces channels to one single channel, and it is a stimulus-independent approach which can be used for different mental tasks. The proposed method is applied to a BCI experiment involving six healthy subjects for classifying five mental tasks, that is, resting state, mental arithmetic, motor imagery of left and right hand, and visual imagination of letter “A.” This study is a part of a research project to build a daily use BCI system for disabled people [32, 33]. A pairwise and a multiclass classification were performed using the two commonly used classifiers, that is, Linear Discriminant Analysis (LDA) and Support Vector Machines (SVM), to find the most suitable task pair for BCI. Performance of our method is also compared with the two existing methods which are commonly used to extract the features for EEG classifications [34, 35]. This paper is organized as follows.  Section 2 gives a brief description of data acquisition including the experimental design and data preprocessing.  Section 3 presents the proposed feature extraction approach and Section 4 gives a short description of the data classification. In Section 5, the results of the experiment are shown along with a discussion.  Section 6 concludes the whole study and proposes ideas for future work.

## 2. Data Acquisition

### 2.1. Experiment and Data Acquisition

#### 2.1.1. Subjects

Six healthy, right-handed subjects (one female) with a mean (standard deviation, SD) age of 30.5 (14.4) participated in the experiments. Except Subject 1, none of the subjects have any experience in BCI experiments. All subjects provided written informed consent before the experiments. All procedures performed by the subjects were in accordance with the ethical standards of the institutional research committee and with the 1964 Helsinki declaration and its later amendments or comparable ethical standards.

#### 2.1.2. Procedure

The subjects were seated on a comfortable chair in a dim lighted, silent room during the recordings. Before each trial, they were informed about the type of task (resting state, multiplication, right hand, etc.) by auditory cues. During the task, they were required to close their eyes to reduce the artefacts from eye blinking/movements. The sequence of mental and motor tasks was as follows: resting state, mental arithmetic, imagination of right hand movement, imagination of left hand movement, and visual imagination of letter “A” [32, 33, 36]. Each trial lasted 10 seconds and the interval between consecutive tasks was about 3-4 seconds. The first 2 seconds in trial were the task preparation time for the subject. The experiments are comprised of 5 experimental runs of 20 trials each (100 trials per task in total). The details of each task are provided below:

- *Resting state (RS)*: the subjects were asked to sit and relax as much as possible without thinking anything.
- *Mental arithmetic task (MA)*: the subjects were given a two-digit multiplication problem to solve in mind without vocalizing or any movement (e.g., 24 × 76 = ?). The problems were not repeated. After the trial, the subject verified whether he reached the solution or not.
- *Right hand imagination task (RH)*: the subjects were told to imagine right hand movement.
- *Left hand imagination task (LH)*: the subjects were required to imagine left hand movement.
- *Letter “A” imagination task (LA)*: the subjects were told to imagine the letter “A” in their mind.

#### 2.1.3. Recordings

EEG data were recorded from the subjects during the experiment, using a 64-Channel Biosemi ActiveTwo EEG system with Ag/AgCl electrodes [32, 33]. The electrodes were placed according to the international 10–20 electrode placement system using Cz as the reference. The grounding electrodes CMS and DRL were mounted on the back of the head. The EEG signals were sampled at 512 Hz.

#### 2.1.4. Channel Selection

We selected 9 channels from different brain regions, that is, frontal (F3/4), central (C3/4), parietal (P3/4, Pz), and occipital (O1/2) areas, for signal analysis. Feature extraction and classification were performed at each single channel.

### 2.2. Data Preprocessing

**Figure 1:**
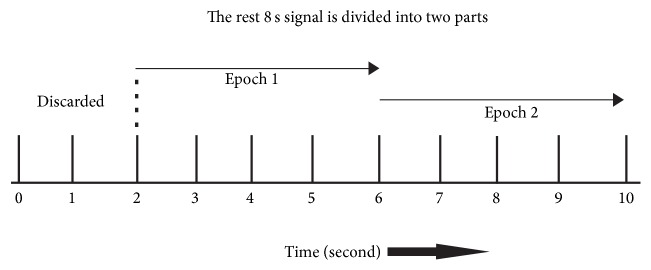
Data segmentation. In each trial, the first 2-s task preparation period was excluded and the remaining 8-second signal was divided into two parts, which resulted in 100 × 2 epochs total for each task.

The first 2-second task preparation period was excluded from the entire 10 seconds in each trial. The remaining 8-second signal was divided into two parts, which resulted in 100 × 2 epochs total for each task (see Figure 1). The EEG signal was filtered using the 10th-order 50 Hz low-pass digital Butterworth filter.

## 3. Feature Extraction

**Figure 2:**
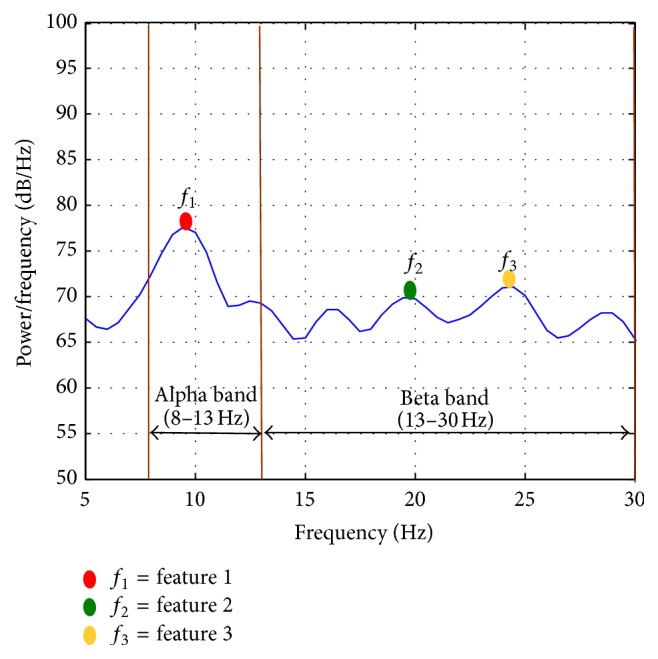
The proposed feature extraction scheme. The curve indicates the PSD of EEG in alpha and beta bands. We selected the highest PSD peak value in the alpha band and the two highest PSD peak values in the beta band as the features.

The general idea of feature extraction is that the high dimensional input data are transformed into a reduced representation set of features while containing the relevant information from the input data. Among feature extraction methods, power spectral density (PSD) analysis is a commonly used method as it extracts the frequency characteristics of signals which enable the detection of mental and motor tasks [4]. Most of the previous studies used this method for investigating epileptics and hypnosis [37–41]. Generally, PSD approaches demonstrate the most consistent robustness and effectiveness in extracting the distinctive spectral patterns for accurately discriminating between motor imagery EEGs [42]. Here, we proposed a novel feature extraction method relying on the frequency distributions of the signal's PSD. In this method, we first computed the PSD based on Welch Periodogram: a hamming window of 400 points was used with a 50% overlap between adjacent windowed sections. We visually inspected the whole frequency range for all subjects. Alpha and beta frequencies are important characteristics of normal EEG activity at rest, and any change of these rhythms might be interpreted as a cortical functioning or information processing indication [43, 44]. In line with previous studies [43, 44], we found that there is a stable pattern in the PSD with different amplitudes for all subjects and for all tasks. This biologically phenomenon allows a classification between different mental tasks. Based on this biological phenomenon, we extracted three features from the alpha (8–13 Hz) and beta (13–30 Hz) bands of PSD by searching the local peak values in the alpha and beta bands separately. The first feature is selected as the highest PSD peak value in the alpha band, which is indicated as *f*_1_ in Figure 2. The second and third features are the two highest PSD peak values in the beta band, which are indicated as *f*_2_ and *f*_3_ in Figure 2.

## 4. Classification

In general, classification is defined as assigning a predefined class to each instance. The goal of classification is to accurately predict the target class for each case in the data. Similar to many previous studies on signal classification, we first dealt with a binary classification problem and then extended the study to multiclass cases. LDA and SVM are used for classification, since they have been known to be efficient classifiers for BCI [17].

### 4.1. Linear Discriminant Analysis (LDA)

Linear discriminant analysis (LDA) is one of the popular classification algorithms and has been successfully applied to many pattern recognition and EEG data classification problems [3, 42]. LDA projects the data onto a lower-dimensional vector space such that the ratio of the between-class distance to the within-class distance is maximized, in order to achieve maximum discrimination. The optimal projection can be readily computed by applying the eigendecomposition to scatter matrices. In this study, we implemented a pairwise and a multiclass LDA for classification.

### 4.2. Support Vector Machines (SVM) Classifier

SVM is a strong classifier which has demonstrated its excellent generalization properties in various applications, including BCIs [12, 45]. The basic idea of SVM is to find the optimal separation hyperplane by maximizing the margin. According to [46], general output of a binary SVM classifier can be computed by the following expression:

(1) $$\begin{array}{c} y=sign\left(\;\overset{N}{\underset{i=1}{\sum }}\alpha_{i}y_{i}k\left(\;x_{i},x_{j}\;\right)+b\;\right). \end{array}$$

Here *α*_*i*_ ≥ 0 are Lagrangian multipliers obtained by solving a quadratic optimization problem, *b* is the bias, and *k*(*x*_*i*_, *x*_*j*_) is a kernel function. The most commonly used kernel function is the Gaussian RBF function which is also used in this study and given by

(2) $$\begin{array}{c} k\left(\;x_{i},x_{j}\;\right)=\exp\left(\;\frac{-{\left\|x_{i}-x_{j}\right\|}^{2}}{2\sigma^{2}}\;\right), \end{array}$$

where *σ* is a user defined parameter showing the width of the kernel function.

In this study, different kernel function types such as exponential and base have been tried, and we found that the best results are obtained with RBF function. Therefore, only the result with RBF function is provided in this paper. We implemented the pairwise SVM to multiclass scenario and obtained the classification performances for both problems.

### 4.3. Data Classification

Data from 9 channels were analysed separately in order to express the classification accuracy in single channel. The classification algorithms discriminated the test data of an unknown task between the given two tasks. Each class had 200 epochs. We randomly chose 50 epochs per class as the training dataset and left the rest as the independent testing dataset. The classifiers were trained using the training dataset. In the testing session we randomly picked up 50 epochs from the testing dataset to test the classification performance. This process was repeated 100 times to get the final classification performance with mean classification accuracy.

Multiclass classification was performed for discrimination of five different tasks. The training and testing methodology followed the same steps in binary classification. We first did pairwise classification for each channel. Then the class label of each channel was attained by max-win voting. Moreover, we calculated the classification accuracy (CA) for four channels (F3/4, C3/4). Based on the CA, the final class for that testing data was predicted by means of max-win voting strategy. In order to see the classification performance of different tasks, we also report individual CA for each task.

## 5. Results and Discussion

### 5.1. Feature Extraction Results

**Figure 3:**
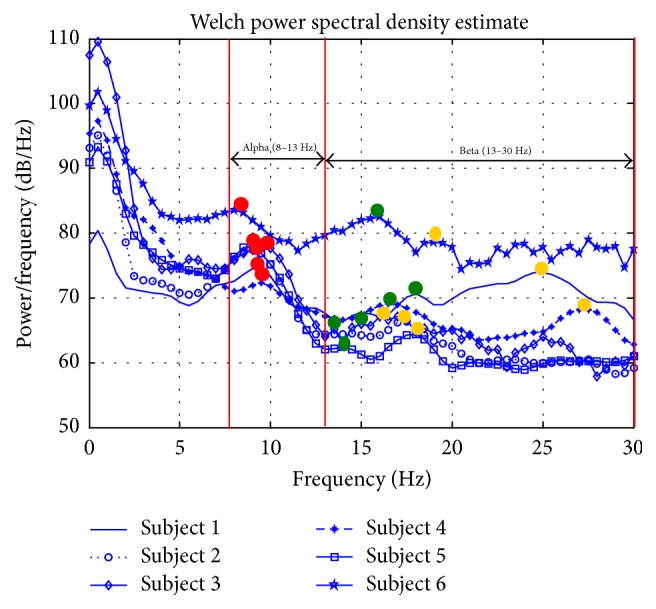
PSD during task RS in channel F3 for all subjects. Red dots indicate feature 1 (the alpha peak), green dots indicate feature 2 (the first peak in the beta band), and yellow dots indicate feature 3 (the second peak in the beta band).

**Figure 4:**
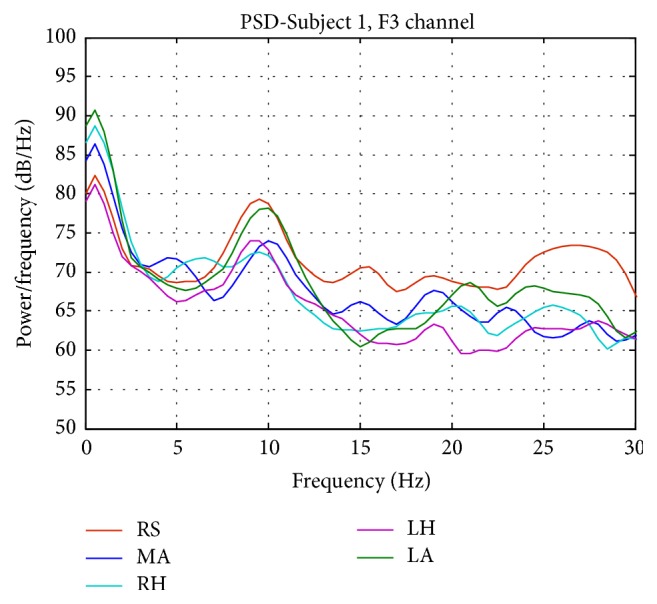
PSD for all five tasks in channel F3 for Subject 1. The difference between tasks is shown in PSD.

The PSD waveforms of six different subjects have consistency in the general characteristics of each mental and motor task.  Figure 3 shows a randomly selected single-trial PSD of all subjects at channel F3 for Task RS. A clear alpha (8–13 Hz) peak is shown for each subject, though there are individual differences of its amplitude. We observed two peaks in beta band, and they have lower amplitude values compared to the peak at the alpha band. The PSD for different tasks at channel F3 for Subject 1 is given in Figure 4. The difference between tasks is shown in PSD, where the alpha peak has the highest amplitude for Task RS. Similar to Task RS, Task LA has higher alpha peak compared to motor imagery tasks (Tasks LH and RH) and Task MA. Beta peaks decrease for the motor imagery tasks compared to other tasks. Rhythmic neural activities within the alpha (8–13 Hz) and beta (13–30 Hz) frequency bands are modulated during imagined mental and motor tasks. Changes in these rhythms provide the neurophysiologic support for extracting the features from the alpha and beta bands. Results from previous studies indicate that alpha wave amplitudes vary with the subject's attention to mental tasks performed with the eyes closed [28, 32, 33]. Beta rhythms are modulated when the subjects are alert and attentive to external stimuli or exert a motor imagery task [28–30]. Specifically, imagination of hand movement typically induces a power decrease in the beta rhythms (namely, event-related desynchronization) over the corresponding functional areas in the sensorimotor cortex [30]. Our findings are consistent with the neurophysiologic knowledge, hence supporting our idea that the selected features would discriminate different mental and motor tasks.

**Figure 5:**
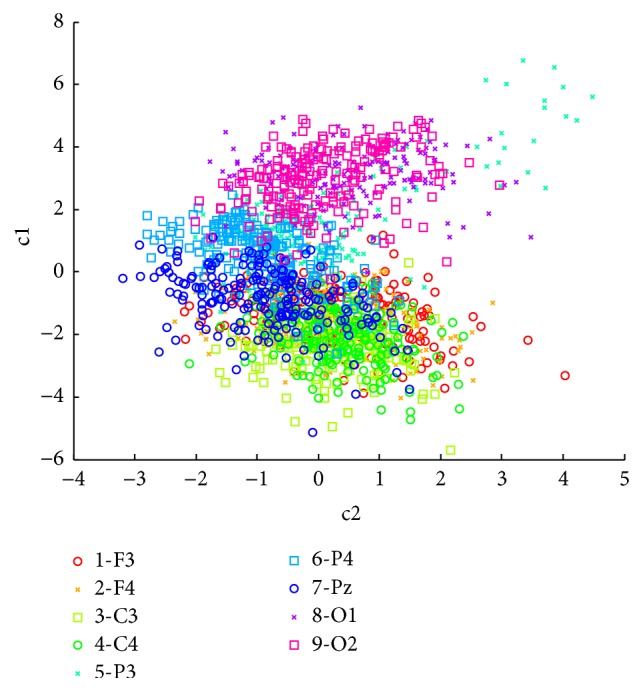
Feature difference between channels for Subject 1. Each channel has different behaviour for the same feature combination.

In order to see whether the selected channels are significantly different from each other on the extracted feature set, a one-way-MANOVA test was performed. The feature difference between channels is shown in Figure 5 for a representative subject. The canonical variables c1 and c2 are linear combinations of the features (a1y in alpha band and a2y and a3y in beta band) for each channel. The null hypothesis (i.e., no difference between channels) is rejected with *p* < 0.05. There was a statistically significant difference between channels based on three features,* d* (estimate of the dimension of the group means) = 2, *p* < 0.005; Wilk's lambda = [0.162; 0.810; 0.99]; and chi-square distribution = [3263; 376; 8]. This difference indicates different discriminative abilities in EEG classification.

**Figure 6:**
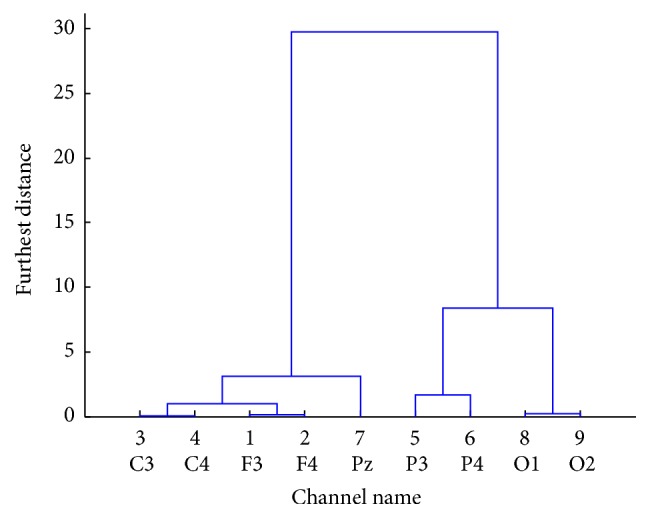
The distances between channels of Subject 1 for RS. A larger distance indicates a big difference between two channels.

In Figure 6, the dendrogram shows the distance between channels. The largest distance was observed between C3 and O1 channels for RS, showing that channels in distant brain regions may carry very different information even for the same task. This result indicates the necessity of channel selection in EEG classification.

### 5.2. Classification Results

Table 1 shows the classification accuracies of all six subjects at the best channel for different task pairs. Using only a single channel, our method can achieve fairly good classification accuracy with SVM (mean accuracy over all tasks and all subject is 83.06%), which indicates the effectiveness of our method in feature extraction and electrode reduction. Although Subject 1 has more experience in using BCI than other subjects, who are naïve to BCI, we did not find significant difference between the performances of Subject 1 and others. As a result, the user's training time can be shortened when using our method. Comparison between using two popular classifiers, that is, SVM and LDA, showed that our method worked better with SVM using a Gaussian kernel.

**Figure 7:**
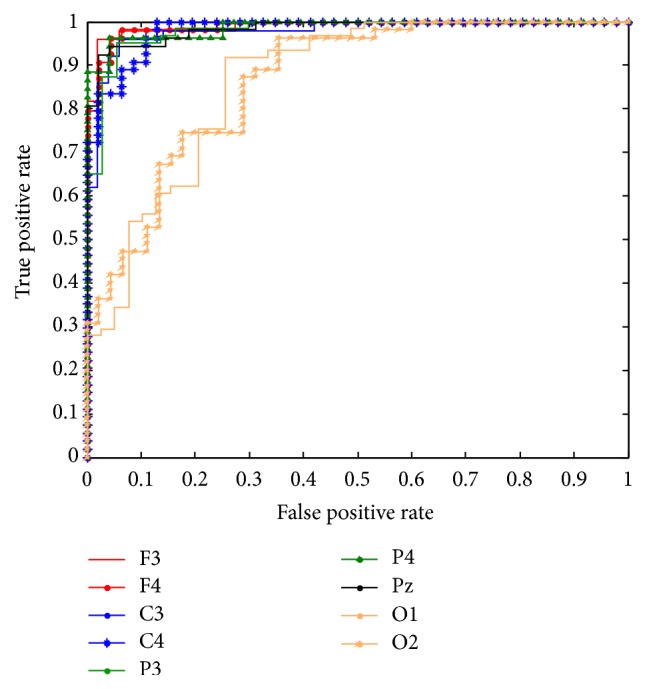
SVM ROC graphs of Subject 1 for 9 channels for RS versus MA. Accuracy is measured by the area under the ROC curve. An area of 1 represents a perfect test; an area of 0.5 represents a worthless test.

Additionally, we examined the plot of sensitivity (true positive rate) versus 1-specificity (false positive rate), namely, receiver operating characteristics (ROC) curve and the area under the curve (AUC) [47] for evaluating the reliability of classification procedure. A sample graph for the performance of Subject 1 on RS versus MA is given in Figure 7, where the point (0,1) indicates the perfect classification. We can see that classification performance from channel F4 (red line in Figure 7) is in the upper left corner of the ROC graph. This result is in line with Tables 1 and 3, where F4 is the best channel for this task pair and this subject, indicating reliability of our classification procedure.

From Table 1, we also can see that the best channel varies with the different task pairs for the same subject. For example, for subject 1 when using SVM as the classifier, the best channel for RS versus MA is F4, while for LH versus LA it is P4. A plausible explanation is that different brain regions have different functions that allow performing different tasks. Although there is a common understanding that individual optimization might be needed for finding the best electrode for few channel based BCIs [19], we did find that some brain regions are important for differentiating some tasks. In our experiment, we found that four subjects have the highest performance at the frontal area of the brain (F3 and F4) for RS versus MA, indicating frontal area may be an important region for distinguishing these two tasks. For the pair RS versus LA, the highest classifications (96.5%) are obtained at the parietal (P3) region for four subjects and at central (C4) regions for the remaining two subjects. For motor tasks, mostly the parietal and central region electrodes (P3, Pz, and C4) have the highest classification performance. These findings might help to find a task-related region of interest for placing the electrode for single-channel BCI. Moreover, some biologically inspired models might be developed for understanding the emotional and cognitive brain processes. Finally, the proposed mental task based approach is a kind of stimulus-independent active BCI approach [48], in which the user has more freedom to attain a certain goal, such as neurofeedback systems, gaming applications, and e-learning platforms.

Different subjects have different task pairs for the best classification, indicating that the performance of binary-class BCI can be improved by individual optimization of task pairs. Identifying the best BCI task pairs for binary-class BCI could be useful for user defined application, for example, neurogames. Among different task pairs, the task pair MA versus LA yields the best mean performance (90.29%) and its performance is close to the best one for most subjects except for the first subject (the subject with BCI experience). Thus, this pair might be the best option for binary-class BCI, in particular for naïve subjects. Nonetheless, the lowest accuracy achieved with the worst combination of task pairs, that is, mean accuracy 73% for RH&LH, is still comparable with the results reported by other single-channel BCI studies [49], indicating the effectiveness of our method.

Table 2 shows the comparisons between our method and two other feature extraction methods which are tested by using our data with Gaussian kernel-based SVM classifier. The method proposed in [34] is based on minimum, maximum, mean, and standard deviation of EEG data which tested single-channel performance, while the method in [35] is for extracting band power features of alpha and beta bands. Shown in Table 2, our method outperforms the existing methods with a mean classification accuracy of 83.06%, indicating that the proposed frequency domain features are more effective in single-channel classification.  Table 3 shows the classification accuracy, sensitivity, and specificity values with standard deviations for RS versus MA tasks of Subject 1 with SVM method in order to show the reliability of the binary classification results. Accuracy is the ratio of the sum of true positives and true negatives to the total population which are in accordance with the classification performance.

The multiclass classification was tested with a pairwise and voting strategy [34, 46] using a few numbers of electrodes. The multiclass classification results with SVM are presented in Table 4 with the overall accuracy values. We achieved comparatively high classification results (91.85%). The experimental results of the proposed method from both binary and multiclass classification showed that this method can be performed in the context of BCI research. Nowadays, BCI systems are only used for patients and military purposes, but in the near future, more practical BCI applications like neurogames may take place in our daily life.

## 6. Conclusions

In this study, a new feature extraction method for EEG signals based on biologically inspired frequency domain characteristics is presented, and its application in BCIs based on single channel is demonstrated. The experimental results indicate the interest of our method in improving the classification accuracy, minimizing the number of electrodes required in a BCI, and reducing the computational cost. The findings are consistent with the neurophysiologic knowledge. Comparison with the existing feature extraction methods shows that our method yields better mean performances which are on par with the state-of-art methods, using only a single-channel EEG and with low computational cost. Application of the proposed method for a multiclass classification further indicates the robustness and efficiency of our method. Additionally, the best task pair for designing a binary-class BCI is also concluded for a naïve subject, which is mental arithmetic versus letter imagination. In future, we will combine this method with some existing artefact removal algorithms for real BCI applications based on the single-channel EEG.

## Figures and Tables

**Table 1:** Classification results for the features extracted by our method. The better classification performances are highlighted in bold for each subject and each task pair. The best classification performances among different task pairs are underlined.

Task pairs	Classifiers	Accuracy %						
		Subjects						
		S1	S2	S3	S4	S5	S6	Mean over subjects
RS&MA	LDA	95.10	71.38	64.76	56.12	57.56	70.76	69.25
		(F3)	(F3)	(O2)	(F3)	(O1)	(F4)	
	**SVM**	**98.36**	**84.38**	**68.76**	**89.96**	**80.84**	**87.20**	**84.91**
		(F4)	(F3)	(F3)	(P3)	(O1)	(F4)	

RS&RH	LDA	86.52	61.66	57.08	54.84	56.08	62.2	63.06
		(F3)	(P3)	(O2)	(C3)	(P4)	(F4)	
	**SVM**	**92.40**	**87.56**	**77.00**	**90.12**	**79.00**	**83.80**	**84.98**
		(F4)	(P3)	(C4)	(P3)	(C3)	(O2)	

RS&LH	LDA	88.88	60.0	56.08	59.8	56.16	60.88	63.63
		(C4)	(C4)	(F3)	(P3)	(P4)	(F4)	
	**SVM**	**94.74**	**70.86**	**58.52**	**90.88**	**88.96**	**86.20**	**81.69**
		(C4)	(F3)	(F4)	(P3)	(C3)	(O2)	

RS&LA	LDA	72.88	72.70	60.82	60.76	56.64	62.2	64.33
		(F3)	(P3)	(O2)	(P3)	(C4)	(F4)	
	**SVM**	**87.18**	**96.50**	**64.20**	**91.20**	**87.92**	**89.92**	**86.15**
		(P3)	(P3)	(C4)	(P3)	(P3)	(C4)	

MA&RH	LDA	66.12	57.76	62.42	62.08	64.48	62.88	62.62
		(O2)	(F3)	(O2)	(P3)	(O2)	(O2)	
	**SVM**	**81.08**	**72.08**	**72.36**	**90.44**	**90.24**	**86.24**	**82.07**
		(O2)	(P3)	(F3)	(P3)	(O2)	(O2)	

MA&LH	LDA	68.64	58.56	65.24	57.64	61.32	62.04	62.24
		(P3)	(F3)	(F3)	(P3)	(O2)	(F4)	
	**SVM**	**82.34**	**78.60**	**94.52**	**90.84**	**88.20**	**98.20**	**88.78**
		(O2)	(C4)	(P4)	(P3)	(O1)	(F4)	

MA&LA	LDA	73.42	68.24	60.96	58.56	62.44	66.44	65.01
		(F4)	(P3)	(F4)	(O2)	(P3)	(F4)	
	**SVM**	**84.16**	**94.98**	**87.50**	**90.80**	**91.08**	**93.26**	**90.29**
		(F4)	(P3)	(P4)	(P3)	(O1)	(O2)	

RH&LH	LDA	52.90	55.56	63.72	54.08	53.72	60.96	56.82
		(C4)	(C3)	(F3)	(C4)	(F3)	(O2)	
	**SVM**	**64.18**	**67.92**	**65.62**	**80.88**	**78.48**	**82.56**	**73.27**
		(C4)	(P3)	(Pz)	(C4)	(P3)	(P3)	

RH&LA	LDA	68.24	67.14	63.44	55.48	57.08	61.72	62.18
		(C4)	(F4)	(F4)	(O2)	(O1)	(F4)	
	**SVM**	**76.48**	**96.08**	**60.34**	**80.32**	**77.24**	**90.0**	**80.07**
		(F3)	(P3)	(F4)	(C3)	(P3)	(F4)	

LH&LA	LDA	59.00	71.96	69.46	55.56	56.96	63.52	62.74
		(P4)	(F4)	(F4)	(O2)	(P4)	(P4)	
	**SVM**	**76.80**	**95.32**	**67.66**	**80.20**	**68.44**	**82.00**	**78.40**
		(P4)	(F3)	(C3)	(C4)	(F3)	(Pz)	

**Mean over tasks**	**LDA**	**73.17**	**64.49**	**62.39**	**57.49**	**58.24 **	**63.36**	**63.19**
	**SVM**	**83.77**	**84.43**	**71.65**	**87.56 **	**83.04 **	**87.94**	**83.06**

**Table 2:** Comparison of the mean SVM performance (over all task pairs) of our method with the existing methods [34, 35].

	S1	S2	S3	S4	S5	S6	Mean over subjects
Our method	83.77	84.43	71.65	87.56	83.04	87.94	**83.06**
Min-max-mean-std [33]	71.95	83.27	73.46	70.00	68.11	66.67	72.24
Band power [34]	73.3	83.5	82.2	62.3	67.2	77.0	74.25

**Table 3:** Accuracy, sensitivity, and specificity values with standard deviations for RS versus MA tasks of Subject 1 with SVM method.

	F3	F4	C3	C4	P3	P4	Pz	O1	O2
Accuracy%	96.80 ± 1.66	96.76 ± 1.54	94.17 ± 1.95	92.69 ± 2.42	84.93 ± 3.14	92.44 ± 2.08	91.71 ± 2.17	86.82 ± 2.51	84.31 ± 2.96
Sensitivity%	96.27 ± 2.65	95.11 ± 2.73	93.80 ± 2.74	91.39 ± 3.84	78.60 ± 3.98	90.38 ± 3.15	89.75 ± 2.95	82.00 ± 3.41	80.21 ± 3.90
Specificity%	97.47 ± 1.94	98.66 ± 1.55	94.71 ± 2.78	94.39 ± 3.08	95.46 ± 3.08	94.95 ± 2.68	94.09 ± 3.04	93.77 ± 3.32	90.31 ± 4.28

**Table 4:** Multiclass classification results with 4 electrodes, that is, F3, F4, C3, and C4.

Classifier	RS	MA	RH	LH	LA	Overall accuracy
SVM						
Accuracy%	100	100	87.04	72.66	99.56	**91.85**
Standard deviation	0.00	0.00	3.55	4.31	0.61	1.69
